# Hypokalemic Periodic Paralysis: a case report and review of the literature

**DOI:** 10.1186/1757-1626-1-256

**Published:** 2008-10-21

**Authors:** Benjamin R Soule, Nicole L Simone

**Affiliations:** 1 National Institutes of Health, National Institute of Allergy and Infectious Disease, Bldg 10-CRC, Room 5-3750, 10 Center Drive, Bethesda, MD 20892, USA; 2 National Institutes of Health, National Cancer Institute, Bldg 10-CRC, Room B2-3500, 10 Center Drive, Bethesda, MD 20892, USA

## Abstract

Hypokalemic Periodic Paralysis is one form of Periodic Paralysis, a rare group of disorders that can cause of sudden onset weakness. A case of a 29 year old male is presented here. The patient presented with sudden onset paralysis of his extremities. Laboratory evaluation revealed a markedly low potassium level. The patient's paralysis resolved upon repletion of his low potassium and he was discharged with no neurologic deficits. An association with thyroid disease is well established and further workup revealed Grave's disease in this patient. Although rare, Periodic Paralysis must differentiated from other causes of weakness and paralysis so that the proper treatment can be initiated quickly.

## Case presentation

A 29 year-old Hispanic male with no significant past medical history presented to the emergency room with sudden onset paralysis. The patient had gone to bed at 10 pm with no weakness and awoke at midnight unable to move his upper or lower extremities. The weakness was bilateral and involved both the proximal muscles of the shoulders and hips as well as the distal extremities. He had no respiratory or swallowing difficulty and was able to move his neck and facial muscles. He denied any pain or paresthesia. Prior to this episode, the patient had been healthy and denied any recent diarrhea, chest pain, shortness of breath, or weight change. He did report several episodes of waking from sleep with a "racing heart." He did not take any medications and denied use of alcohol or drugs, or significant changes in diet or activity levels. His mother had been diagnosed with hypothyroidism but his parents and brother had no history of similar episodes and no other significant illnesses.

On physical exam, the patient's heart rate was 124 and blood pressure was 193/81. He was mildly obese, but otherwise normal in overall appearance. His skin was cool and dry, and the oral mucosa was moist. No jugular venous distension, goiter or lymphadenopathy were appreciated. Cardiac exam revealed tachycardia with a regular rhythm and no murmurs. Examination of the lungs and abdomen were unremarkable. There were no deformities or edema of the extremities and distal pulses were present and equal bilaterally. Neurologic exam revealed flaccid paralysis of all extremities which involved the proximal and distal muscles and included the hips and shoulders. Sensation was intact but deep tendon reflexes were slightly diminished to 3 out of 4 throughout. Cranial nerve function was grossly intact.

**Figure 1:**
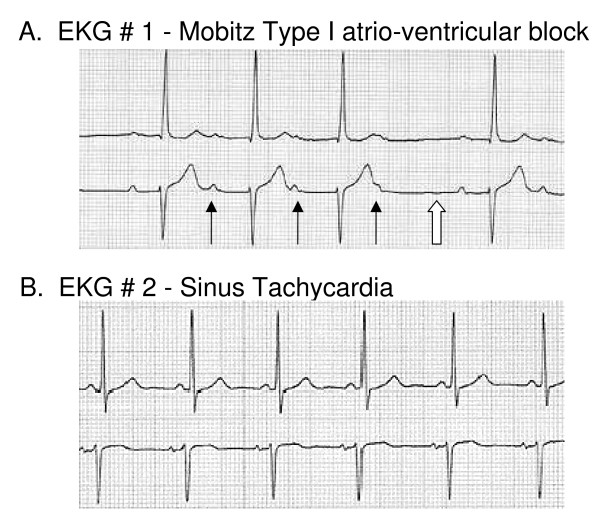
**Progression of EKG Findings**. (A) The initial EKG revealed Mobitz Type I heart block. Several p-waves (solid arrows) are present that are not associated with a QRS complex. The duration of the PR interval continues to increase until a QRS is skipped (open arrow). (B) After treatment, the EKG revealed a return to a sinus rhythm.

Routine chemistry, liver enzymes and complete blood count were normal except for a potassium level of 1.6 (3.5–5 mmol/L). Electrocardiogram revealed sinus tachycardia with Mobitz Type 1 atrio-ventricular block (Figure 1).

Two hours after initiation of intravenous potassium replacement, the patient's neurologic symptoms had completely resolved. His blood pressure remained elevated at 174/86, however repeat electrocardiogram revealed a normal sinus rhythm and rate. Follow up studies were performed to determine the etiology of the patient's hypokalemia. Urine sodium and potassium, and serum aldosterone and renin levels were measured to rule out adrenal involvement and were found to be normal. Thyroid stimulating hormone (TSH), triiodothyronine (T3) and thyroxine (T4) levels were obtained and revealed a markedly abnormal TSH of 0.01 (0.3–5.5 IU/mL) with elevated T3 of 300 (65–164 ng/dL) and T4 of 3.71 (0.58–1.64 ng/dL). Iodine-123 thyroid scan subsequently demonstrated an asymmetric enlargement of the right lobe of the thyroid gland with significantly increased uptake at 2 and 24 hours.

The patient was diagnosed with Hypokalemic Periodic Paralysis associated with Grave's Disease and was started on methimazole for control of his underlying hyperthyroidism and a beta-blocker for control of blood pressure and tachycardia. He was discharged home with an appointment to follow up with an endocrinologist.

## Discussion

Weakness is a common, albeit non-specific, presentation in both the emergency and outpatient setting. Although the differential diagnosis for the complaint of weakness is extensive (Table 1), the focus is considerably narrowed when a patient presents with a demonstrable decrease in muscle strength on physical exam. Strokes and tumors causing nerve compression are potentially life-threatening and must be ruled out first. Other relatively common neurologic concerns include post-ictal paralysis or one of the various motor neuron diseases. Diagnosis of these disorders requires obtaining a complete history with special consideration of timing, duration, and distribution of symptoms. Periodic Paralysis is often overlooked in the initial work-up.

**Table 1:** Causes of acute weakness

**Neurologic**
Stroke
Post-seizure paralysis
Myasthenia gravis
Cataplexy
Multiple sclerosis

**Inflammatory**

Polymyositis
Dermatomyositis

**Infectious**

Polio
Diphtheria
Botulism

**Metabolic**

Porphyria
Alcohol/Opiates
Electrolyte disorders

There are several types of Periodic Paralysis associated with metabolic and electrolyte abnormalities. Of these, Hypokalemic Periodic Paralysis (HPP) is the most common with a prevalence of 1 in 100,000 [1]. The clinical features of the syndrome vary somewhat depending on the underlying etiology but the most striking feature is the sudden onset of weakness ranging in severity from mild, transient weakness to severe disability resulting in life-threatening respiratory failure. Attacks may be provoked by stress such as a viral illness or fatigue, or certain medications such as beta-agonists, insulin or steroids. A perturbation of sodium and calcium ion channels results in low potassium levels and muscle dysfunction [2]. As this is primarily a problem with muscle contraction rather than nerve conduction, tendon reflexes may be decreased or absent but sensation is generally intact. Although the serum potassium level is often alarmingly low, other electrolytes are usually normal. Indeed, total body potassium is actually normal with the change in the serum level reflecting a shift of potassium into cells [3]. Electrocardiographic changes are common, but unlike patients who are truly potassium depleted (Table 2), the changes do not correlate well with the measured serum level [4]. Diagnosis between paralytic episodes is difficult as the patient may have normal strength and potassium levels. Electromyography reveals abnormalities in some patients but is often normal, especially between episodes when no clinically detectable weakness is present.

**Table 2:** Causes of Hypokalemia

**Potassium Depletion – Renal**
Increased aldosterone
Diuretics
Hypomagnesemia
Renal Tubular Acidosis (Type I and II)
Metabolic alkalosis
Liddle's syndrome

**Potassium Depletion – Extra-renal**

Decreased intake
Vomiting/Diarrhea
Zollinger-Ellison Syndrome
Fistulas

**Potassium Shift into Cells**

Increased insulin
Alkalosis
Thyrotoxic Periodic Paralysis
Familial Hypokalemic Paralysis

HPP occurs in several settings and the diagnosis may require an extensive search for the underlying etiology since the treatment varies according to the cause. HPP may occur sporadically in the form of Familial Hypokalmic Paralysis (FHP), a poorly understood disorder which may occur spontaneously or as the result of autosomal dominant inheritance [1]. This form of Periodic Paralysis is felt to be the result of disordered cellular potassium regulation perhaps due to sodium or calcium channel abnormalities [2,5]. Mutations of the *CACNA1S *and *SCN4A *genes have been identified that cause abnormalities in sodium channels resulting in abnormal potassium ion flux. Acute paralytic episodes are treated with potassium replacement and close monitoring of the cardiac rhythm and serum potassium levels. Spironolactone and acetazolamide have been used for prophylaxis with some success although long-term potassium supplementation may be necessary [2].

Thyrotoxic Periodic Paralysis (TPP) occurs in the setting of hyperthyroidism. It is the most common form of HPP and is seen primarily in Asian males occurring in 1.9% of Japanese hyperthyroid patients overall and up to 8% of hyperthyroid Japanese men [6,7]. Hispanic males are also at risk and several cases have been reported [8]. The clinical features are similar to those seen with other forms of HPP, but also include the symptoms of thyrotoxicosis such as weight loss, tachycardia, and anxiety. In patients who develop HPP, however, the symptoms of hyperthyroidism are often quite mild and may be overlooked [4,9]. Paralytic episodes often occur at night, as was the case with this patient [9]. Any cause of hyperthyroidism can be associated with TPP but Grave's disease is the most common. The major feature distinguishing TPP from other Periodic Paralyses is the association of paralytic episodes with the hyperthyroid state. Paralytic episodes can be induced in these patients by administering insulin and glucose, but only when they are hyperthyroid [3]. Euthyroid patients are typically free from spontaneous and induced attacks. The underlying mechanism is not known but is thought to be different from that of FHP since, in that disorder, thyroid hormone levels are normal and the administration of exogenous thyroid hormone does not result in paralytic episodes. Furthermore, the genetic abnormalities felt to be responsible for FHP have not been identified in patients with TPP [5]. Although acute paralytic episodes are treated with potassium replacement, prophylactic potassium or acetazolamide administration is not felt to benefit these patients since potassium levels are normal between episodes and may result in dangerous hyperkalemia [10]. Beta-blocking agents may prevent attacks but the definitive treatment is correction of the underlying thyrotoxicosis [3].

Rarely, HPP can result from substantial gastrointestinal or renal potassium losses. In these cases, total body potassium is depleted and requires aggressive replacement. Endocrine abnormalities such as hyperinsulinemia and primary hyperaldosteronism have been associated with HPP [11]. Surgical removal of the aldosterone producing tumor is the preferred treatment although symptoms can often be managed with spironolactone.

Hyperkalemic Periodic Paralysis and Paramyotonia Congenita are rare forms of Periodic Paralysis that are also associated *SCN4A *mutations that cause gain-of-function abnormalities in the sodium channel resulting in prolonged muscle cell excitation [12]. As a result these conditions worsen with repetitive activity and, in some cases, exposure to cold. Patients often have paralysis of the facial muscles and lower extremities are less affected. Most patients do not require treatment but are instructed to avoid paralysis-inducing situations. There may be some benefit of mexiletine which makes muscle tissue less sensitive to nerve impulses.

Anderson Tawil syndrome is a rare, autosomal dominant disorder that is caused by mutation of the *KCNJ2 *gene in 60% of cases [12]. Mutation of this gene alters the structure and function of potassium channels disrupting the flow of potassium ions in muscle cells leading to Periodic Paralysis and long QT syndrome. Acetazolamide may prevent the paralytic episodes and antiarrhythmics or beta-blockers may prevent ventricular ectopy, but there is little data available.

## Conclusion

This patient presented with sudden onset paralysis and markedly abnormal potassium, TSH, T3 and T4 levels but no significant symptoms of hyperthyroidism. This presentation is typical of TPP. The paralysis resolved completely following potassium replacement and he began a course of methimazole prior to being discharged from the hospital. At the time of discharge, he had no neurologic findings and a normal blood pressure of 126/88 and pulse of 68. He has not suffered any further episodes of paralysis and his TSH is now in the normal range.

Periodic Paralysis is important to consider when seeing a patient with sudden onset weakness or paralysis, especially those with no history or evidence of other diseases and no significant risk factors for stroke. Failure to properly diagnose and treat Periodic Paralysis can be fatal, but rapid correction of potassium abnormalities can resolve the symptoms quickly and completely. When possible, the underlying cause must be adequately addressed to prevent the persistence or recurrence of paralysis.

## Abbreviations

TSH: Thyroid Stimulating Hormone; T3: Triiodothyronine; T4: Thyroxine; HPP: Hypokalemic Periodic Paralysis; FHP: Familial Hyperkalemic Paralysis; TPP: Thyrotoxic Periodic Paralysis

## Competing interests

The authors declare that they have no competing interests.

## Authors' contributions

BPS examined and treated the patient during his hospital course including ordering and interpreting laboratory testing and was a major contributor to writing the manuscript. NLS was a major contributor to writing the manuscript. All authors have read and approved the final manuscript.

## Consent

Written informed consent was obtained from the patient for publication of this case report and accompanying images. A copy of the written consent is available for review by the Editor-in-Chief of this journal.
